# Field study to determine the reliability of HIV viral load results shows minimal impact of delayed testing in South Africa

**DOI:** 10.4102/ajlm.v13i1.2364

**Published:** 2024-05-28

**Authors:** Diana R. Hardie, Howard Newman, Joanna Reid, Nei-Yuan Hsiao, Gert van Zyl, Lucia Hans, Jasantha Odayar, Stephen Korsman

**Affiliations:** 1 Department of Pathology, Faculty of Health Sciences, University of Cape Town, Cape Town, South Africa; 2 National Health Laboratory Service, Cape Town, South Africa; 3 National Health Laboratory Service, Port Elizabeth, South Africa; 4 Division of Medical Virology, Faculty of Health Sciences, Stellenbosch University, Cape Town, South Africa; 5 National Health Priority Programme, National Health Laboratory Service, Johannesburg, South Africa; 6 Department of Molecular Medicine, Faculty of Health Sciences, University of the Witwatersrand, Johannesburg, South Africa; 7 Division of Epidemiology and Biostatistics and the Centre for Infectious Disease Epidemiology and Research, Faculty of Health Sciences, University of Cape Town, Cape Town, South Africa

**Keywords:** HIV viral load stability, delayed testing, plasma preparation tubes, high-throughput viral load testing, diagnostic accuracy

## Abstract

**Background:**

Understanding factors that impact HIV viral load (VL) accuracy in resource-limited settings is key to quality improvement.

**Objective:**

We evaluated whether testing delay and specimen storage between 25 °C and 30 °C before testing affected results.

**Methods:**

Between November 2019 and June 2023, 249 individuals on antiretroviral therapy, or with newly diagnosed HIV, were recruited from clinics in Cape Town and Gqeberha, South Africa, and three plasma preparation tubes were collected. One tube was tested within 24 h, while the others were stored uncentrifuged at ambient temperatures before testing. Centrifugation and testing of matched samples were performed on Day 4 and Day 7 after collection.

**Results:**

Time delay and ambient storage had minimal impact in specimens with a Day 1 VL of > 100 copies/mL. When grouped by Day 1 VL range, 96% – 100% of specimens at Day 4 and 93% – 100% at Day 7 had VLs within 0.5 log copies/mL of the first result. The greatest variability at Days 4 and 7 was observed when the Day 1 VL was < 100 copies/mL. However, there was no trend of increasing difference over time. Of Day 1 specimens with undetectable VL, or VL < 50 copies/mL, 80% had concordant results at Day 4 and 78% at Day 7.

**Conclusion:**

These results show that VL is stable in plasma preparation tubes for 7 days when stored at room temperature. There is significant variability in specimens with low VL, but variability is not affected by testing delay.

**What this study adds:**

Ideal HIV VL testing conditions are frequently unachievable in resource-limited settings. Data are needed on whether this impacts on the validity of test results. Our results provide reassurance that storage at ambient temperature for up to 7 days before testing does not substantially affect the VL result.

## Introduction

Viral load (VL) testing is an essential tool for monitoring patients on anti-retroviral therapy (ART) and, at a programmatic level, provides data to assess the Joint United Nations Programme on HIV/AIDS 95:95:95 targets,^1^ specifically the third target which defines the proportion of patients on ART that are virologically suppressed. South Africa has the largest ART treatment programme in the world, with > 6 million people on therapy.^2^ In 2022, more than 6 m VL tests were performed as part of this programme.^3^ South Africa’s VL testing model involves centralised testing in high-throughput laboratories. Major challenges that have the potential to compromise test accuracy relate to logistics and pre-analytical problems, which include sub-optimal specimen collection, transport, storage, handling, and testing delay.

Currently, VL platform manufacturers advise that plasma should be separated within 6 h to 24 h of collection, then stored at 4 °C or frozen at −80 °C if not tested within 5 days.^4,5^ In resource-constrained environments, specimens frequently reach testing sites beyond the recommended time after collection and may be subjected to further delays before testing takes place. The extent to which these pre-analytical factors compromise the accuracy of results has been evaluated in laboratory simulation studies,^6,7^ and also a systematic review.^8^ Findings suggest that viral RNA is preserved beyond the currently recommended testing time. Good preservation of viral RNA was also found in a local study performed at Groote Schuur Hospital (GSH) using routine diagnostic specimens collected in ethylenediaminetetraacetic acid tubes or ethylenediaminetetraacetic acid-plasma preparation tubes (PPT). In this study, diagnostic specimens were stored after initial HIV VL testing at a range of times and storage temperatures and then re-tested.^9^ The VL in specimens stored for up to 1 week had very good concordance with the first result.^9^ However, tubes had already been centrifuged prior to storage and the study was performed entirely in the laboratory environment. In addition, it was only possible to follow trends in VL from the same patient over 2 time points due to specimen volume constraints. More studies are therefore needed to gain a better understanding of the factors that affect VL test reliability, as this knowledge is critical for improving the standard of testing in resource constrained environments, like South Africa. To expand on our previous findings, a field study was undertaken where participants were recruited to provide additional specimens for VL testing. By means of serial testing of specimens from the same participants we evaluated the extent to which testing delay and adverse storage (including storage temperature and centrifugation) affects the reliability of VL results.

## Methods

### Ethical considerations

Ethical approval for the study was obtained from the Human Subjects Research Ethics Committee of the Faculty of Health sciences at the University of Cape Town (HREC Ref 159/2019). Written informed consent was obtained from all participants prior to enrolment. Only individuals of 18 years or older were recruited. Study-related data were stored on a password-protected device to which only the principal investigator had access.

### Study objectives

The study objectives were as follows: to evaluate the impact of delayed testing on the reliability of HIV RNA quantification in diagnostic specimens, collected and stored in un-centrifuged PPT tubes and to describe the impact of storage at a warmer ambient temperature (storage between 25 °C and 30 °C) in diagnostic specimens in un-centrifuged PPT tubes. We also evaluated whether centrifugation (enabling physical separation of plasma from cells) prior to storage improves the concordance of results where testing is delayed (evaluated at one site, namely GSH).

### Study design

The study was performed at three sites within the National Health Laboratory Service network of HIV VL testing laboratories. Patients were recruited from four ART clinics, three in the Cape Metro and one in the Eastern Cape Province of South Africa. Enrolment for two sites (Gqeberha/Port Elizabeth [PE] and Tygerberg Hospital [TBH]) ran from 21 November 2019 to 24 January 2020 for PE, and 05 December 2019 to 23 January 2020 for TBH. Enrolment for the third site (GSH) began on 28 August 2022 and ended on 06 June 2023 (following normalisation of laboratory services after the severe acute respiratory virus-coronavirus 2 pandemic).

Patients who were due to have a VL test or who were newly diagnosed with HIV and not yet on therapy, were approached, and consented to give two extra blood tubes (5 mL – 7 mL per tube) for HIV VL testing. Agnostic patient recruitment was not done as we were not trying to collect a sample set reflective of the HIV-positive population in South Africa, but rather wanted to evaluate a technical issue in stored specimens. Thus, we aimed to collect roughly half of the specimens from patients with low-level or undetectable viral loads (on ART) and half from patients who were expected to have unsuppressed VLs (not yet started on antiretroviral drugs).

**FIGURE 1:**
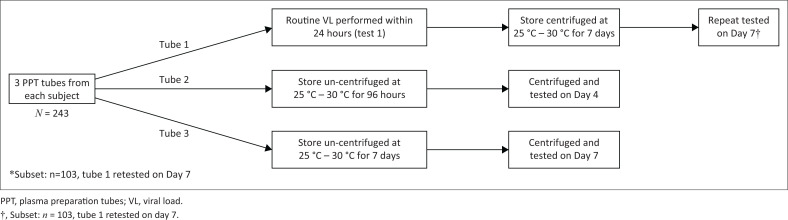
Study plan for testing factors affecting accuracy of viral load samples, South Africa, 2019–2023.

Three PPT tubes were collected during the same blood draw and delivered to the regional HIV VL testing laboratory (Figure 1). On arrival, one tube was centrifuged (at approximately 3000 g for 10 min) and sent for immediate HIV VL testing. Testing was completed within 24 h of sample collection. The result from this test was issued for routine patient management. The other two tubes were stored uncentrifuged in routine field conditions where the temperature varied between 25 °C and 30 °C. Four days after collection, one of the stored tubes was centrifuged (at 3000 g for 10 min) and a VL test was performed. Similarly, 7 days after collection, the third tube was centrifuged and a VL test was performed. Viral load testing was performed using the routine assay in each of the three participating laboratories at the time of study, namely Roche CAP/CTM (TBH laboratory), Roche 6800 (PE laboratory) (both Roche Molecular Systems, Pleasanton, California, United States) and Abbott Alinity m (GSH laboratory; Abbott Laboratories, Chicago, Illinois, United States). On a subset of patients (103 specimens, done at GSH laboratory only), HIV VL was repeated at Day 7 on the tube that had been centrifuged and tested on Day 1. The Day 1 centrifuged tubes were stored under the same conditions as the yet untested ones, prior to repeat testing. The tubes were not re-centrifuged prior to repeat testing on Day 7. They were used to assess whether storage in an already centrifuged state improved stability of VL in the sample.

### Data analysis

Data analysis was performed using Microsoft Excel (Microsoft Corporation, Redmond, Washington, United States). Bland-Altman plots were used to evaluate differences in Day 1 versus Day 4 VL results and between Day 1 and Day 7.^10^ Viral load values were log transformed as this is the standard way of expressing these values in clinical practice.

For analysis, unless otherwise specified, samples with a VL that was lower than the limit of detection (LDL) were assigned a value of ‘1’ (0 log) copies/mL. Results with a VL value of < 20 copies/mL were assigned a value of ‘19’ (1.28 log) copies/mL, otherwise the log value of the reading in copies/mL was used for analysis. We considered a difference in VL of < 0.3 log (or 2-fold difference) to reflect ‘no change’ as this is within the expected variability of this technology. A change of > 0.5 log copies/mL (> 3-fold difference) was considered likely to reflect a change that was caused by factors other than random expected variation.^11^ In addition, the results were evaluated in terms of whether the results obtained at Day 4 or Day 7 resulted in a reclassification of a patients’ VL control status. For this analysis: a viral load of ≤ 0 copies/mL was considered to reflect viral suppression;^12,13^ a viral load of 50 copies/mL–1000 copies/mL was considered to reflect low-level viraemia (LLV);^12,13^ and a VL value of > 1000 copies/mL was considered to reflect unsuppressed VL or viral failure.^12,13^

## Results

A total of 249 participants were consented and provided 3 PPT tubes for testing (51 from TBH, 60 from Gqeberha (PE) and 138 from GSH. Details from each study site are given in Table 1. Specimens from 6 patients arrived at the laboratory more than 24 h after collection and these were excluded from the study. Altogether, 123 participants were already on ART and 120 were newly diagnosed as HIV-positive, but not yet on ART.

**TABLE 1:** Characteristics of patients and testing sites used for viral load test accuracy study, South Africa, 2019–2023.

Site	Patients recruited (*n*)	Patients on ART (*n*)	Newly diagnosed patients (*n*)	VL technology used for testing
PE	60	30	30	Roche 6800
TBH	51	25	26	Roche CAP/CTM
GSH	138†	64	68	Abbott Alinity m

ART, anti-retroviral therapy; PE, Port Elizabeth (Gqeberha); TBH, Tygerberg Hospital; GSH, Groote Schuur Hospital; VL, viral load.

† , Six recruited patients were excluded from the analyses, because their samples arrived at the laboratory more than 24 h after collection.

Day 1 VLs were segregated into the following VL categories: 55 (22.6%) were LDL; 46 (18.9%) were detectable, but < 50 copies/mL; 7 (2.9%) were 50 copies/mL – 100 copies/mL; 28 (11.5%) were 2–3 log copies/mL; 30 (12.3%) were 3–4 log copies/mL; 36 (14.8%) were 4–5 log copies/mL; 30 (12.3%) were 5–6 log copies/mL; and 11 (4.5%) were > 6 log copies/mL (Table 2).

**TABLE 2:** Proportion of samples tested on Day 4 and Day 7 within 0.3 log copies/mL and 0.5 log copies/mL of Day 1 viral load value, by Day 1 VL category, South Africa, 2019–2023.

Day 1 VL by category	Samples (*n*)	Day 4 VL		Day 7 VL	
		Within 0.3 log of Day 1 result (%)	Within 0.5 log of Day 1 result (%)	Within 0.3 log of Day 1 result (%)	Within 0.5 log of Day 1 result (%)
LDL	55	51	51	60	60
< 2 log	53	51	60	55	62
2–3 log	27	89	96	81	93
3–4 log	30	87	97	87	97
4–5 log	36	81	100	86	97
5–6 log	30	93	97	97	97
> 6 log	11	73	100	91	100

LDL, lower than the limit of detection; VL, viral load.

Applying current guidelines to define the participants’ HIV control status, as determined by the Day 1 VL value, 101 participants would have been classified as virally suppressed, 35 had LLV and 107 had unsuppressed VLs (Table 3). For reference, the full data set containing the matched Day 1, Day 4, and Day 7 VL results for each patient is available as Online Supplementary Document 1.

**TABLE 3:** Viral load concordance on Day 4 and Day 7 according to viral control status as determined by the Day 1 test, South Africa, 2019–2023.

Virus control status on Day 1 test	Number	Day 4		Day 7	
		Percentage concordant (%)	Number mis-classified	Percentage concordant (%)	Number mis-classified
Viral suppression (LDL to < 50 copies/mL)	101	81	19†	79	21‡
Low-level viraemia (50 copies/mL to 1000 copies/mL)	35	86	5§	86	5§
Unsuppressed (> 1000 copies/mL)	106	96	4¶	96	4¶
3–4 log	30	87	4¶	87	4¶
4–5 log	35	100	0	100	0
5–6 log	30	100	0	100	0
> 6 log	11	100	0	100	0

LDL, limit of detection.

† , patients with this status on Day 1 would have been misclassified as having low-level viraemia (*n* = 17) or virological failure (*n* = 2) had testing been conducted on Day 4;

‡ , patients with this status on Day 1 would have been misclassified as having low-level viraemia (*n* = 19) or virological failure (*n* = 2) had testing been conducted on Day 7;

§ , patients classified as having low-level viraemia on Day 1 would have been misclassified as having virological failure (*n* = 3) or viral suppression (*n* = 2) had testing been conducted on Day 4 and Day 7;

¶ , all patients with this Day 1 status would have been misclassified as having low-level viraemia had testing been conducted on Day 4 or Day 7.

### Viral load concordance at Days 1, 4 and 7

**FIGURE 2:**
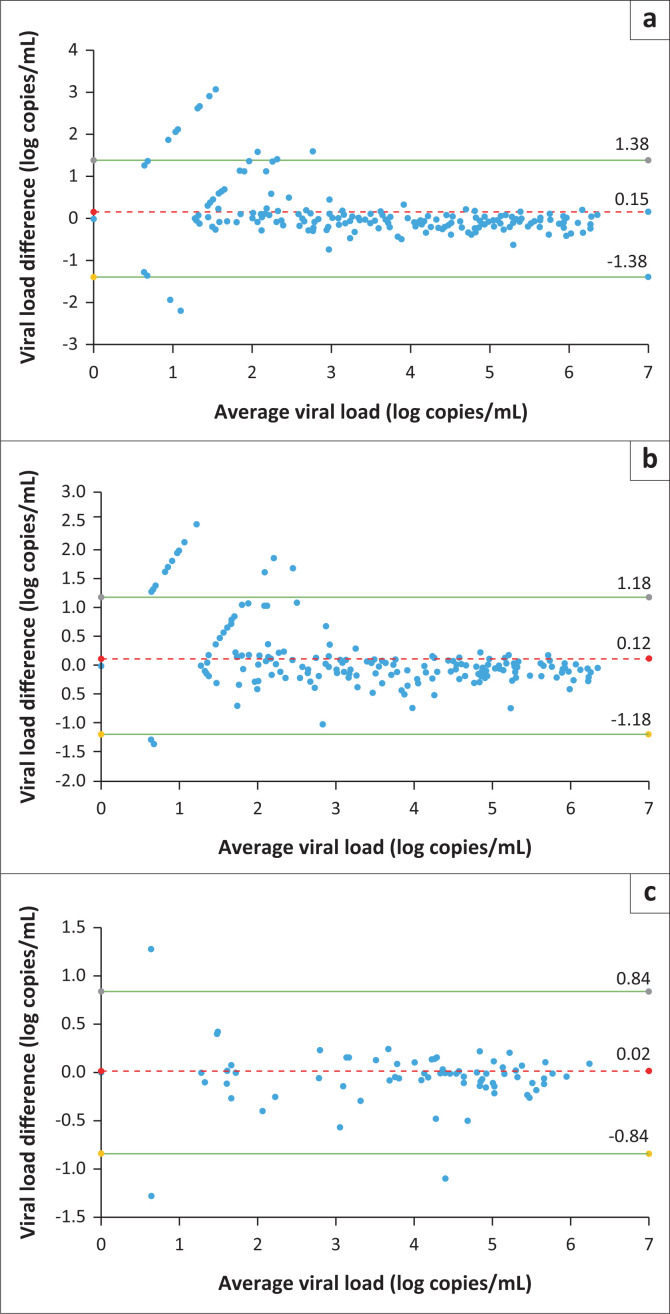
Variability of all paired viral load results at Days 4 and 7, South Africa, 2019–2023. Bland-Altman plots of Day 1 versus Day 4 VL (a) and Day 1 versus Day 7 (b) show that viral load measurements tended to be systematically higher by an average of 0.12 log at Day 4 and 0.14 log at Day 7 as compared to Day 1. The bias tended to be greater in samples with low RNA levels (< 2 log copies/mL); (c) shows variability of paired viral loads of Day 1 samples that were re-run (R) on Day 7, with the Bland Altman plot revealing minimal bias and even lower variance in Day 1 versus Day 7 results. Green lines represent +2 and −2 standard deviations and the red dotted line represents the mean difference in HIV viral load on the Bland Altman plots.

Overall, there was very little difference in paired readings at both Day 4 and Day 7 compared with Day 1. The mean bias at Day 4 was 0.15 and for Day 7 was 0.12 log copies per mL, indicating a higher VL reading in the samples where testing was delayed (Figure 2). For samples with an initial VL of > 2 log copies/mL, the paired VL value at Day 4 and Day 7 was within 0.5 log copies/mL in 96% – 100% of instances for Day 4 and 93% – 100% of instances for Day 7 (Table 2), signifying minimal impact of the testing delay on the quantification. The greatest variation was observed for specimens with Day 1 VLs less than 2 log copies/mL.

When differences in paired VLs were evaluated using important thresholds that define clinical response, namely viral suppression (VL undetectable or < 50 copies/mL), LLV (VL 50 copies/mL – 1000 copies/mL) and viral failure/unsuppressed VL (> 1000 copies/mL) (Table 3), similarly good concordance was observed, with most specimens at Days 4 and 7 having VL in the same clinical range as Day 1. Lowest agreement was in the 101 samples with a Day 1 VL below 50 copies/mL, but nonetheless 81% remained < 50 copies/mL at the Day 4 test and 79% at the Day 7 test. Most of the discordant results, 17 specimens at Day 4 and 19 specimens at Day 7, shifted into the LLV category, while for 2 specimens at Day 4 and 2 specimens at Day 7, the VL was > 1000 copies/mL.

Concordance was higher, at 86% in specimens that were LLV on Day 1 testing at both Days 4 and 7, and even higher, at 96% in specimens where the Day 1 VL was > 1000 copies/mL.

Patients with a Day 1 VL that was detectable, but < 50 copies/mL, were 2.6 times more likely to have a detectable VL at Day 4 and 3.4 times more likely to have a detectable VL at Day 7 than patients with an undetectable Day 1 VL (LDL).

Overall, detection in a later specimen appeared to be stochastic. There was no trend to suggest that this phenomenon worsened with time. Its frequency appeared to be affected by testing site and technology used, as it varied from 0% (GSH, Abbott Alinity), 11% and 22% at Days 4 and 7 (TBH, Roche CAP/CTM) to 50% (PE, Roche Cobas 6800).

### Viral load concordance in samples centrifuged on Day 1

Storage of specimens already centrifuged appeared to improve concordance. The Day 7 VL values in specimens that were stored in a centrifuged condition (plasma separated from the cells) were very similar to the Day 1 value (Figure 2c). The mean difference in VL between Day 1 and Day 7 was 0.03 log copies/mL and standard deviation was narrower, at 0.42, than for the specimens that were first centrifuged and tested on Day 7. Only one result had a clinically significant difference (–1.1 log copies/mL) in VL between Day 1 (4.95 log copies/mL) and Day 7 (3.85 log copies/mL).

## Discussion

In this field study conducted in Cape Town and Gqeberha in South Africa between 2019 to 2023, we found that delay of HIV VL testing for up to 7 days after specimen collection and storage at ambient temperature had minimal effect on test results. These findings are compatible with previous work done in South Africa in 2017, using stored laboratory specimens,^9^ and also support the findings of a systematic review by Bonner et al.^8^ The study conditions were designed to match more closely the real-world situation in low- and middle-income countries with high HIV disease burdens and large ART programmes, where testing is often delayed and storage temperatures are higher than recommended due to logistical issues. These results provide reassurance that time delay and ambient storage is not a major cause of result inaccuracies.

Most of the variability that resulted in misclassification of a patient’s status was observed in specimens with Day 1 VLs of < 2 log copies/mL (from virally suppressed patients and those with LLV). In particular, a proportion of results in the viral suppression range shifted into the LLV range at one or both later time points, and some that were LLV shifted into the viral suppression range. The most probable explanation for this is the inherent variability of real-time polymerase chain reaction technology in specimens with target levels close to the limit of detection.^14^ In such specimens, target may be only intermittently detected in test replicates, and greater variability can be expected in the quantified value.^15^ Of note is that specimens from patients that were virally suppressed, but had a detectable VL that was < 50 copies/mL at Day 1, were 2 times to 3 times more likely to have a detectable VL in a stored specimen than if the VL was undetectable at Day 1. Low-level viraemia reflects a state of incomplete viral suppression and has been associated with increased risk of VL failure in the future.^16,17,18^ Because of this, there is a move to set the VL indicative of viral failure (currently defined as > 1000 copies/mL) at a lower VL threshold, such as at 200 or 50 copies/mL. As can be seen in this study, this can be expected to increase the chance of misclassification of patients due to the inherent variability of real-time polymerase chain reaction in this range. Confirmation of viraemia on a second specimen would help to mitigate this and is recommended in current guidelines.^12,13^

Occasional specimens displayed a higher-than-expected variability, namely 4 that were virally suppressed on Day 1, but > 1000 copies/mL at either Day 4 or Day 7. It is probable that factors other than time delay were responsible. Potential explanations for this stochastic variability could be specimen contamination with HIV RNA during processing, inadequate centrifugation prior to testing or some other undefined laboratory factor.^19,20,21^ Technical factors clearly played a role in this study, as the proportion of specimens that were LDL on Day 1, but detectable later, varied markedly at the different testing sites.

One additional measure to improve test concordance that was evaluated was centrifugation of specimens prior to storage. In a subset of specimens, the same tube that was centrifuged and tested on Day 1, was rerun on Day 7. These results showed very low variability and no specimen had a clinically actionable change in VL when comparing the Day 1 and Day 7 results. A caveat is that this experiment was only done at the site where there was very low variability in the stored specimens anyway.

### Limitations

Only three specimens were collected from each patient, and this limited the number of factors we could evaluate. Testing occurred across three sites and different testing platforms could have accounted for some of the differences that were seen, for example a much lower rate of detectable viral RNA in later specimens with Day 1 suppressed VL at one site. These will need to be evaluated at a later stage. Specimens were only tested once on Day 1. A repeat test on Day 1 would have provided key data on the inherent variability of VL results on our platforms and given context to the variability observed at the later time points.

### Conclusion

Our field study provides further evidence that time delay has minimal impact on VL quantification when specimens are stored at room temperature for up to 7 days. This extends the time allowable before VL testing needs to be done. Most of the discordant results were in the LLV range and likely reflect inconsistent detection at the limit of detection of the assay. Centrifugation of specimens prior to storage improves the concordance of results at later time points and early centrifugation after collection should be prioritised for HIV testing programmes. The contribution of other patient-related and technical issues requires further investigation.
